# An Investigation of Fractional Bagley–Torvik Equation

**DOI:** 10.3390/e22010028

**Published:** 2019-12-24

**Authors:** Azhar Ali Zafar, Grzegorz Kudra, Jan Awrejcewicz

**Affiliations:** 1 Department of Automation, Biomechanics and Mechatronics, Lodz University of Technology, 1/15 Stefanowskiego St., 90-924 Lodz, Poland; grzegorz.kudra@p.lodz.pl (G.K.); jan.awrejcewicz@p.lodz.pl (J.A.); 2 Department of Mathematics, Government College University, Lahore 54000, Pakistan

**Keywords:** Caputo derivative, fractional calculus, linear oscillator, analytic solutions

## Abstract

In this article, we will solve the Bagley–Torvik equation by employing integral transform method. Caputo fractional derivative operator is used in the modeling of the equation. The obtained solution is expressed in terms of generalized *G* function. Further, we will compare the obtained results with other available results in the literature to validate their usefulness. Furthermore, examples are included to highlight the control of the fractional parameters on he dynamics of the model. Moreover, we use this equation in modelling of real free oscillations of a one-degree-of-freedom mechanical system composed of a cart connected with the springs to the support and moving via linear rolling bearing block along a rail.

## 1. Introduction

The concept of fractional calculus (FC) and entropy are very important for the investigation of complex dynamical systems and hence got the attention of researchers, physicists and mathematicians. Machado [1] investigated the importance of entropy for the analysis of complex dynamical systems. Furthermore, Lopes and Machado [2] used the FC tools for the study of complex systems. Ubriaco [3] proposed the entropy functions based on FC for the analysis of dynamical systems. Prehi et al. in [4] and Luchko [5] discussed the entropy in non-integer order diffusion processes. For more about FC and entropy we refer [6,7,8]. The non-local nature of fractional derivatives allows to describe changes in an interval. This important property makes these derivatives suitable to simulate more physical and complex phenomena. For more details, we refer the readers to [9,10,11]. As, FC is nearly as old as the standard integral and differential calculus with a long list of applications. Since, the non-integer order derivative parameter used in the modelling of dynamical systems behaves as the rheological parameter and influence the properties of the dynamical systems, it is seen that different interdisciplinary problems can likewise be solved with good accuracy by the aid of non-integer order derivatives [12].

Atanackovic and Stankovic [13] have investigated the motion of a viscoelastic bar with non-integer order derivative type of dissipation under time dependent loading. Fa [14], for clear physical interpretations adopted the non-integer order derivatives in the problem relating to free falling bodies. In viscoelasticity, the first application of FC is seem to be done by Bagley and Torvik [15,16] whereas, Makris et al. [17] approximated the applicable value of non-integer order parameter in the non-integer order derivative model that has good compliance with the material properties of the material and the experimental results. In addition, for the display of the linear response regime, one dimensional viscoelastic models with fractional order generalizations are proven to be very good [18] and in accordance with the second law of thermodynamics. Lazopoulos [19] introduces FC in the continuum mechanics and introduced the non-local constitutive relations. Likewise, Carpinteri et al. [20] have proposed a fractional approach to non-local mechanics. So, list of the applications of FC is too long to be added here. Despite of the usefulness of the FC, the major criticism regarding the use of fractional derivatives is the inability of fractional derivatives to behave like derivatives. As, they failed to correspond to differentials and unable to satisfy the requirements of differential topology for being derivatives [19]. However, most of the known fractional derivatives have only operative character instead of a derivative one.

To remove this drawback, Lazopoulos [19], proposed the fractional L-derivative but it again does not satisfy the conditions of differential topology. Lately, Lazopoulos [21] formulated the ∧-fractional derivative, a modification of the fractional L-derivative. This ∧-fractional derivative behaves like classical derivative rules. Moreover, in [22] K. A. Lazopoulos and A. K. Lazopoulos solved the fractional bending problem using the ∧-fractional derivative.

In 1983, Bagley and Torvik [15] formulated an equation to study the viscoelastically damped structures, later in [16] they used this equation to investigate the behaviour of real material using FC. This equation called Bagley–Torvik equation (BTE) plays a vital role in a large number of applied science and engineering problems. More specifically, any linearly damped fractional oscillator with damping term has fractional derivative of order 1.5 can be represented by BTE. Particularly, the equation with half or one and the half order derivative can predict the models with materials where damping depends on frequency. It can also describe motion of real physical systems, the modeling of the motion of a rigid plate immersed in a viscous fluid and a gas in a fluid respectively [9,23].

The form of BTE [9] is

(1) $$\lambda_{2}\frac{d^{2}u\left(t\right)}{dt^{2}}+\lambda_{1}D_{t}^{\frac{3}{2}}u\left(t\right)+\lambda_{0}u\left(t\right)=f\left(t\right);\;\;\;\;t>0,$$

with u(0)=0,$u^{\prime }\left(0\right)=0,$ where $\lambda_{2}\neq 0$, the mass of the thin rigid plate immersed in the viscous fluid, $\lambda_{1}=2A\sqrt{\mu \rho }$ is the constant depending on the area of the plate immersed, density and viscosity of the fluid, $\lambda_{0}$ is the spring’s stiffness, f:[0,∞]→R is a given function denoting the applied force to the plate and u(t) represents the motion of the plate. $D_{t}^{\frac{3}{2}}$ is the non integer order differential operator in Caputo sense [9] of order $\frac{3}{2}$. The existence and uniqueness of solutions to such fractional differential equations(FDEs) and related analytical results have been presented in [9].

The importance of BTE motivated the researchers to show interest in its solutions. For example Podlubny [9] obtained the numerical solution of the BTE with the aid of fractional Green’s function. Numerical analytical solutions of the equation were developed adopting the Adomian decomposition method [23,24,25] and hybridisable discontinuous Gelerkin method [26]. Enesiz, Keskin, and Kurnaz in [27] proposed a new algorithm called generalized Taylor collocation algorithm for solving the BTE. Diethelm [28] reformulate the equation into first order coupled FDE and solve the model with Adam predictor and corrector approach. Wang and Wang [29] have studied the solution of the BTE with half-order and one and the half order derivatives. Ghorbani and Alavi [30] used He’s variational iteration method for the solution of BTE.

More recently, Bansal and Jain [31] discussed analytical solution of BTE by a generalized differential transform method, Anjara and Solofoniaina [32] solved the equation by Adomian’s method, Fazli and Nieto [33] proved the results for the existence and approximations of the solutions of BTE. Gamel et al. [34] used the Chelyshkov–Tau approach for solving BTE. Moreover, Uddin and Ahmad [35] formulated the numerical scheme, while Setia et al. [36] obtained the solutions of BTE by using second kind Chebyshev wavelet.

It is important to note that, while solving the FDE numerically, for example using differential transform method, Adomian method or even in generalized differential transform method etc., first we have to fix the value of the fractional order parameter then we solve the problem, but the beauty of Laplace transform method is that one has the provision to consider the arbitrary value of the fractional order parameter for obtaining the result and finally that non integer order parameter becomes the rheological parameter. It gives us the liberty to conveniently choose the value of the parameter so that the theoretical results are in accordance with the experimental result. Moreover, this parameter helps us in the validation of our results with the existing classical results.

With these motivations, our aim is to solve the BTE in its most general form. The solution of the BTE will be presented involving Lorenzo–Hartley generalized G function [37]. Further, the agreement of our results with the exiting solutions as well as the control of the non-integer order parameter on the motion of the plate is shown by graphical representations. Further, we use this equation in modelling of real free oscillations of a one-degree-of-freedom mechanical system composed of a cart connected with the springs to the support and moving via linear rolling bearing block along a rail.

## 2. Preliminaries

The fractional integral is defined as [9]

$$I_{t}^{\alpha }g\left(t\right)=\frac{1}{\Gamma (\alpha )}\int_{0}^{t}\frac{g(\tau )}{{\left(t-\tau \right)}^{1-\alpha }}d\tau ;\;\;\;\;\;0<\alpha <1.$$

The fractional order derivative in the sense of Caputo is defined as [9]

$$D_{t}^{\alpha }g\left(t\right)=\frac{1}{\Gamma (1-\alpha )}\int_{0}^{t}\frac{g^{\prime }\left(\tau \right)}{{\left(t-\tau \right)}^{\alpha }}d\tau ;\;\;\;\;\;0<\alpha <1$$

and $D_{t}^{\alpha }g\left(t\right)=g^{\prime }\left(t\right)$ when α=1.

The Laplace transform of this fractional derivative operator is defined as

$$L\left[D_{t}^{\alpha }g\left(t\right)\right]=q^{\alpha }G\left(q\right)-q^{\alpha -1}g\left(0\right)-q^{\alpha -2}g^{\prime }\left(0\right).$$

The fractional order derivative in the sense of Riemann–Liouville is defined as [9]

$${}^{RL}D_{t}^{\alpha }g\left(t\right)=\frac{1}{\Gamma (1-\alpha )}\frac{d}{dt}\int_{0}^{t}\frac{g(\tau )}{{\left(t-\tau \right)}^{\alpha }}d\tau ;\;\;\;\;\;0<\alpha <1$$

and ${}^{RL}D_{t}^{\alpha }g\left(t\right)=g^{\prime }\left(t\right)$ when α=1.

Moreover, we can write ${}^{RL}D_{t}^{\alpha }g\left(t\right)=\frac{d}{dt}\left(I_{t}^{1-\alpha }g\left(t\right)\right).$

The ∧-fractional derivative is defined as [21]

$${}^{\wedge }D_{t}^{\alpha }g\left(t\right)=\frac{dI_{t}^{1-\alpha }g\left(t\right)}{dI_{t}^{1-\alpha }t};\;\;\;\;\;0<\alpha <1,$$

the ∧-fractional derivative behaves as traditional derivative with local properties. For more information on ∧-fractional derivative, we refer to [21,22].

## 3. General Form of the Bagley–Torvik Equation and Its Solution

The Bagley–Torvik equation in generalized form is written as

(2) $$\lambda_{2}D_{t}^{\beta }u\left(t\right)+\lambda_{1}D_{t}^{\alpha +1}u\left(t\right)+\lambda_{0}u\left(t\right)=f\left(t\right);\;\;\;\;t>0,$$

where 1<β<2 and 0<α<1,

Subject to

(3) $$u\left(0\right)=u_{0},\;\;u^{\prime }\left(0\right)=u_{1},$$

with $u_{0}$ and $u_{1}$ are real numbers.

Applying Laplace transform [38] and using initial conditions, we obtain

(4) $$\lambda_{2}\left(q^{\beta }\bar{u}\left(q\right)-q^{\beta -1}u\left(0\right)-q^{\beta -2}u^{\prime }\left(0\right)\right)+\lambda_{1}\left(q^{\alpha +1}\bar{u}\left(q\right)-q^{\alpha }u\left(0\right)-q^{\alpha -1}u^{\prime }\left(0\right)\right)+\lambda_{0}\bar{u}\left(q\right)=\bar{f}\left(q\right)$$

or

(5) $$\bar{u}\left(q\right)=\frac{u_{0}\lambda_{2}q^{\beta -1}+u\left(0\right)\lambda_{1}q^{\alpha }+\lambda_{2}u_{1}q^{\beta -2}+u_{1}\lambda_{1}q^{\alpha -1}+f\left(q\right)}{\lambda_{2}q^{\beta }+\lambda_{1}q^{\alpha +1}+\lambda_{0}},$$

where *q* is the Laplace transform parameter.

Using formula $\frac{1}{x+a}=\sum_{k=0}^{\infty }\frac{{\left(-1\right)}^{k}x^{k}}{a^{k+1}}$, last expression can be rewritten as

(6) $$\begin{array}{c} \bar{u}\left(q\right)=\sum_{k=0}^{\infty }\frac{{\left(-1\right)}^{k}\lambda_{1}^{k}}{\lambda_{2}^{k+1}}(\frac{u_{0}\lambda_{2}q^{(\alpha +1)k+\beta -1}}{{\left(q^{\beta }+\frac{\lambda_{0}}{\lambda_{2}}\right)}^{k+1}}+\frac{u_{0}\lambda_{1}q^{(\alpha +1)k+\alpha }}{{\left(q^{\beta }+\frac{\lambda_{0}}{\lambda_{2}}\right)}^{k+1}}+\\ +\frac{u_{1}\lambda_{2}q^{(\alpha +1)k+\beta -2}}{{\left(q^{\beta }+\frac{\lambda_{0}}{\lambda_{2}}\right)}^{k+1}}+\frac{u_{1}\lambda_{1}q^{(\alpha +1)k+\alpha -1}}{{\left(q^{\beta }+\frac{\lambda_{0}}{\lambda_{2}}\right)}^{k+1}}+\frac{\bar{f}\left(q\right)q^{(\alpha +1)k}}{{\left(q^{\beta }+\frac{\lambda_{0}}{\lambda_{2}}\right)}^{k+1}}). \end{array}$$

Taking inverse Laplace transform [38], we get

(7) $$\begin{array}{c} u\left(t\right)=\sum_{k=0}^{\infty }\frac{{\left(-1\right)}^{k}\lambda_{1}^{k}}{\lambda_{2}^{k+1}}(u_{0}\lambda_{2}G_{\beta ,(\alpha +1)k+\beta -1,k+1}\left(-\frac{\lambda_{0}}{\lambda_{2}},t\right)+\\ +u_{0}\lambda_{1}G_{\beta ,(\alpha +1)k+\alpha ,k+1}\left(-\frac{\lambda_{0}}{\lambda_{2}},t\right)+u_{1}\lambda_{2}G_{\beta ,(\alpha +1)k+\beta -2,k+1}\left(-\frac{\lambda_{0}}{\lambda_{2}},t\right)+\\ +u_{1}\lambda_{1}G_{\beta ,(\alpha +1)k+\alpha -1,k+1}\left(-\frac{\lambda_{0}}{\lambda_{2}},t\right)+\int_{0}^{t}f\left(t-\tau \right)G_{\beta ,(\alpha +1)k,k+1}\left(-\frac{\lambda_{0}}{\lambda_{2}},\tau \right))d\tau , \end{array}$$

where *G* is the Lorenzo–Hartley “generalized *G* function” and is defined as [37]

$$G_{a,b,c}\left(d,t\right)=\sum_{j=0}^{\infty }\frac{d^{j}\Gamma \left(c+j\right)}{\Gamma \left(c\right)\Gamma \left(j+1\right)\Gamma \left((c+j)a-b\right)}t^{(c+j)a-b-1}$$

and

$$G_{a,b,c}\left(d,t\right)=L^{-1}\left[\frac{q^{b}}{{\left(q^{a}-d\right)}^{c}}\right];\;\;\;Re\left(ac-b\right)>0,|\frac{d}{q^{a}}|<1.$$

## 4. Results and Discussion

In this section, by graphical illustrations, we will testify the agreement of our results with the exiting solutions of BTE obtained by different methods in the literature, and the control of the non-integer order parameter on the model equation.

For example, when $\lambda_{2}=\lambda_{1}=\lambda_{0}=1$, $u_{0}=u_{1}=1$, α=0.5, β=2 and f(t)=t+1 Equation (7) becomes

(8) $$\begin{array}{c} u\left(t\right)=\sum_{k=0}^{\infty }{\left(-1\right)}^{k}(G_{2,\frac{3}{2}k+1,k+1}\left(-1,t\right)+G_{2,\frac{3k+1}{2},k+1}\left(-1,t\right)+G_{2,\frac{3}{2}k,k+1}\left(-1,t\right)+\\ +G_{2,\frac{3k-1}{2},k+1}\left(-1,t\right)+\int_{0}^{t}\left(t-\tau +1\right)G_{2,\frac{3}{2}k,k+1}\left(-1,\tau \right)d\tau ). \end{array}$$

**Figure 1:**
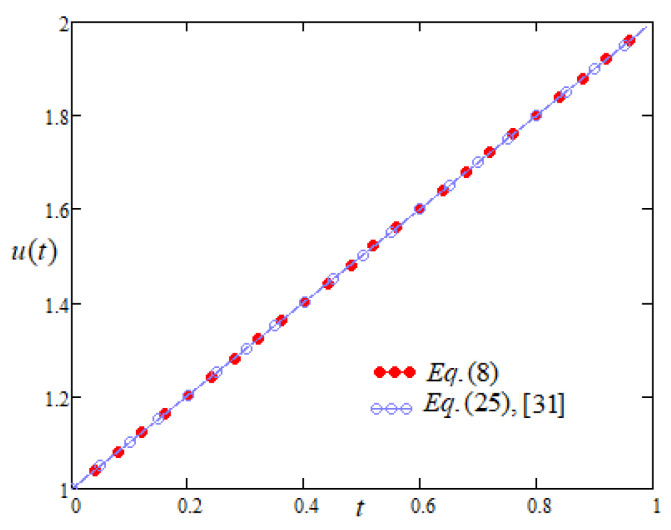
Comparison of the profiles of u(t) versus *t* represented by Equation (8) and Equation (25) of [31].

As evident from Figure 1, it is equivalent to the Equation (25) of Bansal and Jane [31] (obtained by using the improvement of differential transform method) as the profiles of the two solutions overlap each other. Moreover, the profiles of Equation (7) are the same as Exp. (1) of Udin and Ahmad [35] and example B of Setia et al. [36] respectively obtained by using integral representation in complex plane and 2nd kind Chebyshev wavelet.

Similarly, for $\lambda_{2}=\lambda_{1}=\lambda_{0}=1$, $u_{0}=u_{1}=0$, α=0.5, β=2 and $f\left(t\right)=2+4\sqrt{\frac{t}{\pi }}+t^{2}$ Equation (7) takes the form

(9) $$u\left(t\right)=\sum_{k=0}^{\infty }{\left(-1\right)}^{k}\int_{0}^{t}\left(2+4\sqrt{\frac{t-\tau }{\pi }}+{\left(t-\tau \right)}^{2}\right)G_{2,\frac{3}{2}k,k+1}\left(-1,\tau \right)d\tau .$$

**Figure 2:**
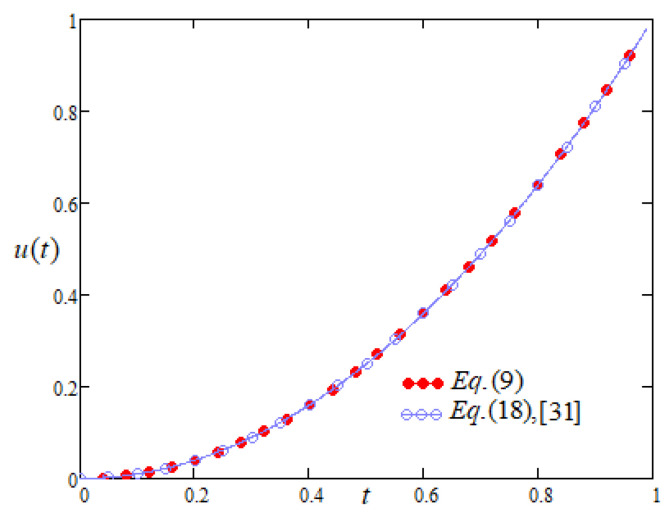
Comparison of the profiles of u(t) versus *t* represented by Equation (9) and Equation (18) of [31].

Profiles of Equation (9) are the same (evident from Figure 2) as obtained by Bansal and Jane (Equation (18), [31]), where the solution was obtained by using generalised differential transform method. Moreover, for $\lambda_{2}=1,$$\lambda_{1}=\frac{2}{5},$$\lambda_{0}=0.25$, $u_{0}=0,$$u_{1}=1$, α=0.5, β=2 and $f\left(t\right)=\frac{1}{4}t^{2}-\frac{1}{4}t-\frac{8}{5}\sqrt{\frac{t}{\pi }}-2$ Equation (7) becomes

(10) $$\begin{array}{c} u\left(t\right)=\sum_{k=0}^{\infty }{\left(-1\right)}^{k}{\left(\frac{2}{5}\right)}^{k}(G_{2,\frac{3}{2}k,k+1}\left(-\frac{1}{4},t\right)+\frac{2}{5}G_{2,\frac{3k-1}{2},k+1}\left(-\frac{1}{4},t\right)+\\ +\int_{0}^{t}\left(\frac{{\left(t-\tau \right)}^{2}-\left(t-\tau \right)}{4}-\frac{8}{5}\sqrt{\frac{t-\tau }{\pi }}-2\right)G_{2,\frac{3}{2}k,k+1}\left(-\frac{1}{4},\tau \right)d\tau ). \end{array}$$

**Figure 3:**
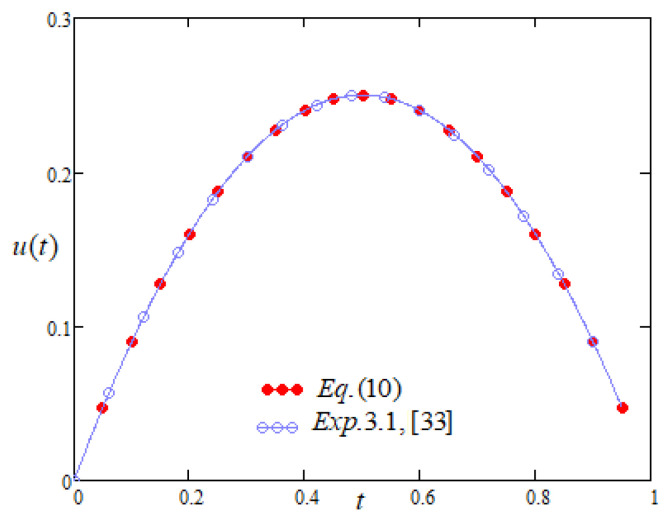
Comparison of the profiles of u(t) versus *t* represented by Equation (10) and Exp. 3.1 of [33].

From Figure 3, Equation (10) is equivalent to the results shown in Exp. (3.1) by Fazli and Nieto [33] by different technique.

Again, for $\lambda_{2}=\lambda_{1}=\lambda_{0}=1$, $u_{0}=u_{1}=1$, α=0.5, β=2 and $f\left(t\right)=t^{3}+\frac{8}{\sqrt{\pi }}t^{\frac{3}{2}}+7t+1$, Equation (7) reduces to

(11) $$\begin{array}{c} u\left(t\right)=\sum_{k=0}^{\infty }{\left(-1\right)}^{k}(G_{2,\frac{3}{2}k+1,k+1}\left(-1,t\right)+G_{2,\frac{3k+1}{2},k+1}\left(-1,t\right)+\\ +G_{2,\frac{3}{2}k,k+1}\left(-1,t\right)+G_{2,\frac{3k-1}{2},k+1}\left(-1,t\right)+\\ +\int_{0}^{t}\left({\left(t-\tau \right)}^{3}+\frac{8}{\sqrt{\pi }}{\left(t-\tau \right)}^{\frac{3}{2}}+7\left(t-\tau \right)+1\right)G_{2,\frac{3}{2}k,k+1}\left(-1,\tau \right)d\tau ). \end{array}$$

**Figure 4:**
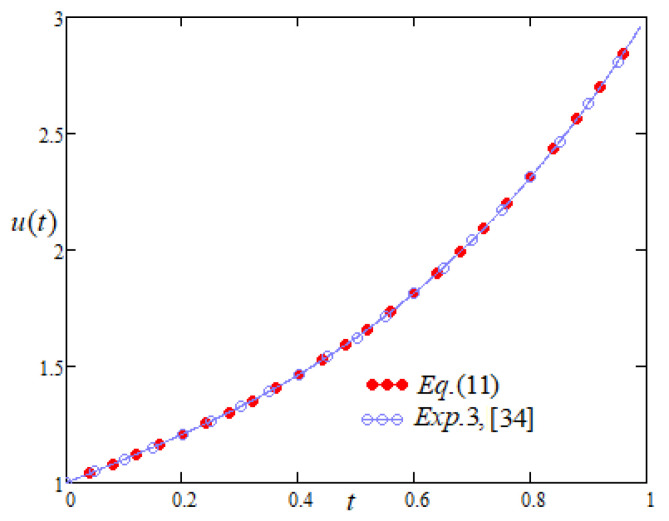
Comparison of the profiles of u(t) versus *t* represented by Equation (11) and Exp. 3 of [34].

From Figure 4, it is noticed that Equation (11) is similar to the results shown in Exp. (3.2) by Gamel et al. [34] by adopting the Chelyshkov–Tau approach for the solution of BTE as the two profiles overlap each other.

From these results, it is verified that our results has good agreement with the previously obtained results by different numerical methods. Hence, obtained results could be used as the exact solutions for the comparison of the solution of the BTE by new numerical simulations and methods.

Next, in order to get more insight about the control of the fractional order parameters α and β on the dynamics of the plate for different modes of the applied force f(t), we discuss the following three cases.

### 4.1. Case-I: When Driving Force on the Plate Is Constant

**Figure 5:**
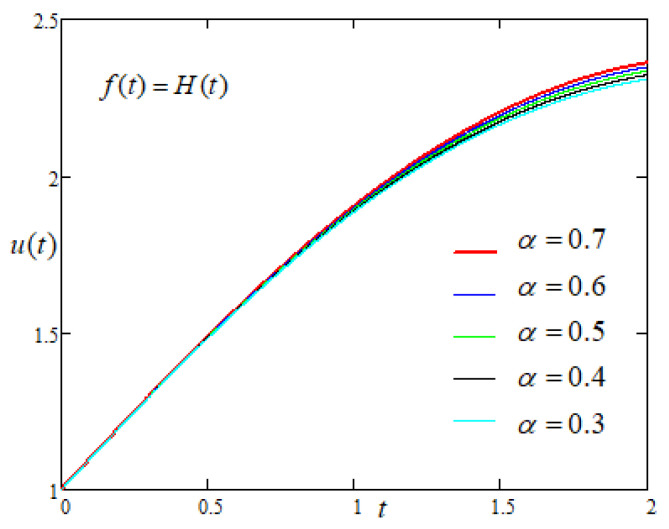
Comparison of the profiles of u(t) versus *t* represented by Equation (7) for several values of α when f(t)=H(t).

**Figure 6:**
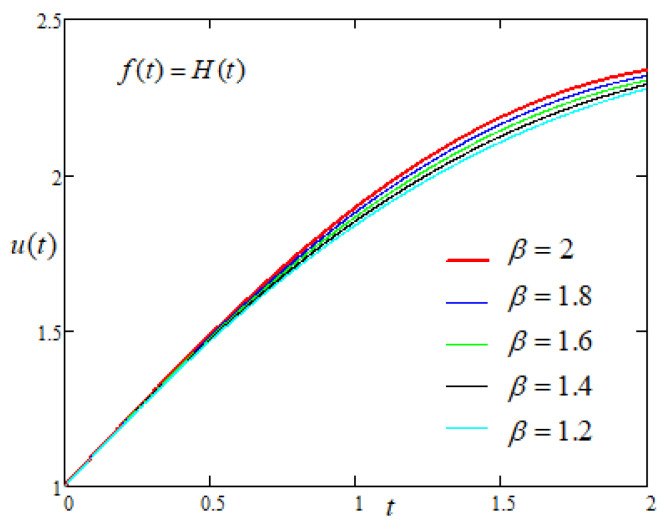
Comparison of the profiles of u(t) versus *t* represented by Equation (7) for several values of β when f(t)=H(t).

In order to study the influence of the non integer order parameters α and β on the motion of the plate with constant driving force of the form f(t)=H(t) applied on the plate, Figure 5 and Figure 6 are prepared and it is noticed that the u(t) increases with the increasing values of fractional parameters.

### 4.2. Case-II: When Driving Force on the Plate Is a Quadratic Function of Time

**Figure 7:**
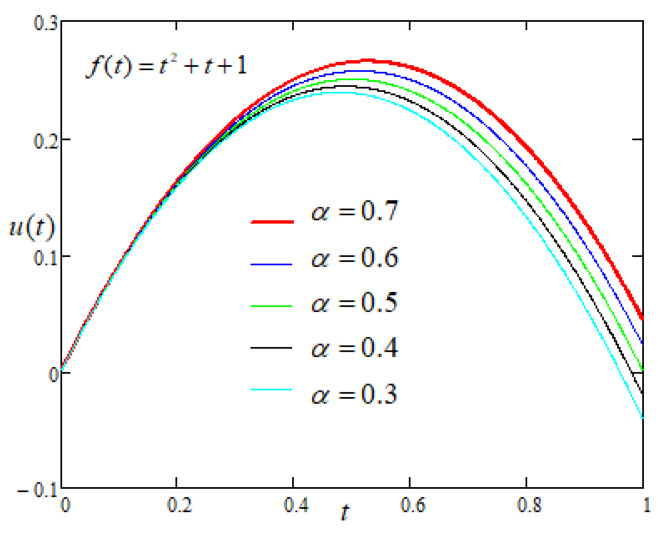
Comparison of the profiles of u(t) versus *t* represented by Equation (7) for several values of α when $f\left(t\right)=t^{2}+t+1$.

**Figure 8:**
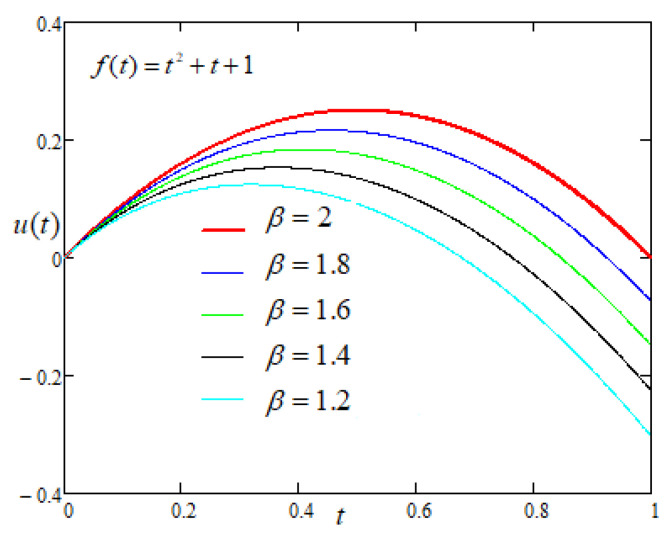
Comparison of the profiles of u(t) versus *t* represented by Equation (7) for several values of β when $f\left(t\right)=t^{2}+t+1$.

Now, to study the influence of the non integer order parameters α and β on the motion of the plate with driving force of the form $f\left(t\right)=t^{2}+t+1$, applied on the plate, Figure 7 and Figure 8 are prepared and same trend is reported as in case-I.

### 4.3. Case-III: When Driving Force on the Plate Is a Periodic Function of Time

**Figure 9:**
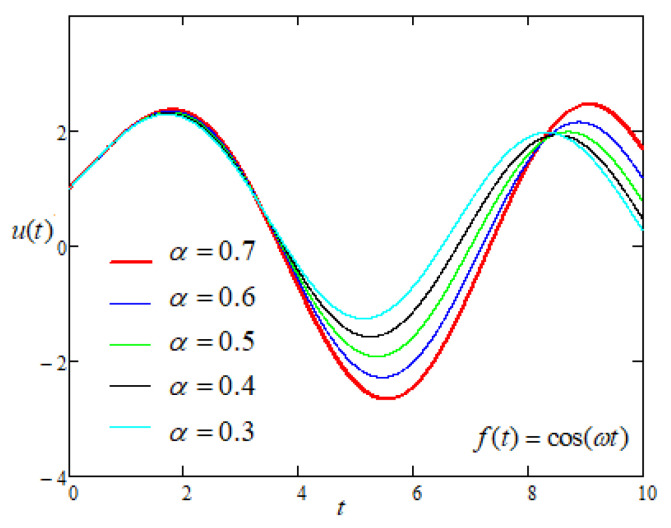
Comparison of the profiles of u(t) versus *t* represented by Equation (7) for several values of α when f(t) = cos(ωt).

**Figure 10:**
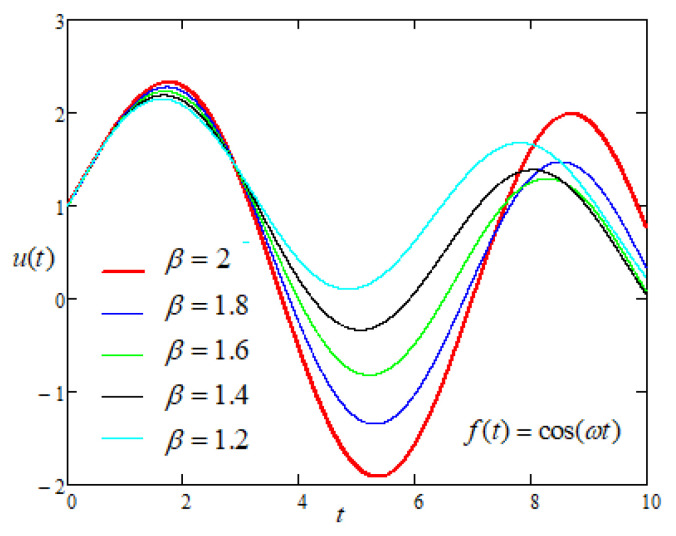
Comparison of the profiles of u(t) versus *t* represented by Equation (7) for several values of β when f(t) = cos(ωt).

Finally, to study the influence of the non integer order parameters α and β on the motion of the plate with sinusoidal driving force of the form f(t) = cos(ωt) applied on the plate Figure 9 and Figure 10 are prepared and it is noticed that the motion of the plate increases with the increasing values of fractional parameters.

From all these Figure 5, Figure 6, Figure 7, Figure 8, Figure 9 and Figure 10 it is noticed that, the influence of fractional parameters is significant and sensitive to the driving force.

## 5. Modelling of Experimental One-Degree-of-Freedom Mechanical Oscillator

In spite of the fact that the original physical interpretation of the BTE is motion of a rigid plate immersed in a viscous fluid, this section is devoted to an attempt of using this equation in modelling of real free vibrations of mechanical system with one-degree-of-freedom composed of a cart connected with the springs to the support and moving via linear rolling bearing block along a rail.

**Figure 11:**
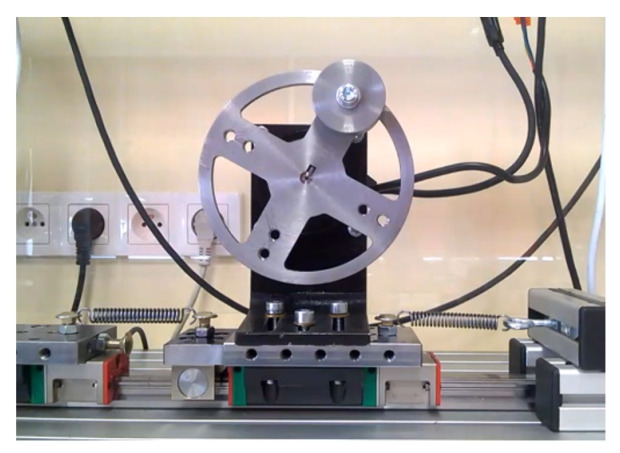
Experimental stand.

This experimental system, presented in Figure 11, is a special case of reconfigurable experimental rig, used for studying mechanical systems of multi-degree-of-freedom with impacts, magnetic springs, and different kinds of forcing [39,40]. Position of the cart is measured by the use of Hall sensors and magnetic tape integrated with the rail. Previous investigations have shown that sum of Coulomb friction and viscous damping is a good model of resistance forces in the rolling bearings. In this work, the extension of the model with partial derivatives will be tested. It is proposed the following mathematical description of free oscillations of a cart

(12) $$\begin{array}{ccc} D_{t}^{\beta }u\left(t\right)+\lambda_{1m}D_{t}^{\alpha +1}u\left(t\right)+\lambda_{0m}u\left(t\right) & = & -T_{0m}sign\left(u^{\prime }\left(t\right)\right)\;\;\;\;if\;\;u^{\prime }\left(t\right)\neq 0\\ & = & \lambda_{0m}u\left(t\right)\;\;\;\;if\;\;u^{\prime }\left(t\right)=0,|\lambda_{0m}u\left(t\right)|<T_{0m} \end{array}$$

and

(13) $$\begin{array}{ccc} D_{t}^{\beta }u\left(t\right)+\lambda_{1m}D_{t}^{\alpha }u\left(t\right)+\lambda_{0m}u\left(t\right) & = & -T_{0m}sign\left(u^{\prime }\left(t\right)\right)\;\;\;\;if\;\;u^{\prime }\left(t\right)\neq 0\\ & = & \lambda_{0m}u\left(t\right)\;\;\;\;if\;\;u^{\prime }\left(t\right)=0,|\lambda_{0m}u\left(t\right)|<T_{0m} \end{array}$$

where $\lambda_{1m}=\frac{\lambda_{1}}{\lambda_{2}}$, $\lambda_{0m}=\frac{\lambda_{0}}{\lambda_{2}}$ and $T_{0m}$ is a parameter corresponding to the constant friction force.

The Equations (12) and (13) are the Bagley–Torvik equation with piecewise constant external force f(t) and the solution is obtained through gluing the segments of the solutions presented in the previous sections and corresponding to different regimes of motion defined in Equations (12) and (13).

**Figure 12:**
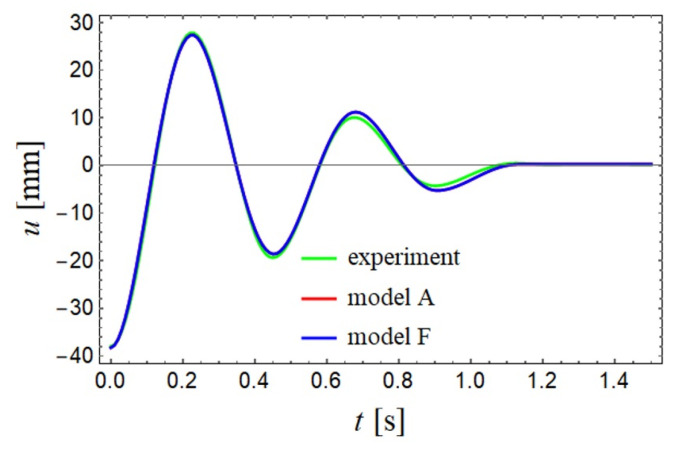
Profiles of the selected versions of mathematical model fitted to the experimental fee oscillations.

The parameters are identified minimizing numerically the objective function

$$F_{O}\left(\alpha ,\beta ,\lambda_{0m},\lambda_{1m},T_{om}\right)$$

defined as average squared difference between the experimental and theoretical displacement of the cart. It is used on experimental free motion of the trolley, with the initial part of the solution cut off, so it starts from the extremum. Initial velocity in the model is assumed to be zero, while initial position is taken from the experimental solution. There are identified parameters of different versions of the model presented in Table 1, where the values of the parameters in italics denote constant values during the identification process. For example model *A* using Equation (12) corresponds to the differential equations of motion with integer derivatives. In the case of model *B* using Equation (13) both the derivatives are non-integer and all the parameters are identified. However, the solution has not been found better than in the case of model *A* (see the corresponding values of the objective function) and the derivatives are almost integer. In the case of models *C*–*F* using Equation (12), there are different tested cases where there are assumed different constant and non-integer values of the derivatives. It is found an interesting feature that the investigated experimental solution can be modelled assuming different values of non-integer derivatives and the final result are almost the same, however the parameters have different values. Figure 12 exhibits solutions to the selected versions (*A* and *F*) of mathematical model fitted to the experimental free oscillations. Since the solutions are very similar, they overlap each other.

## 6. Conclusions and Future Work

In this article the well-known Bagley–Torvik equation is solved by employing integral transform method and examined its validation by comparing them graphically with the existing results in literature as well as the experimental rig of real free oscillations of one degree of freedom mechanical system.

The main features of our general results are:

The obtained solution is expressed in terms of generalized G function, and could be used to recover the results for different values of initial conditions and applied force to the plate. Regarding the control of the fractional parameters, it is reported that the motion of the plate is an increasing function of the fractional parameters and their influence is sensitive to the applied force to the plate. The accuracy of the obtained results is tested by comparing them graphically with the existing results in literature, developed usually by some numerical techniques and the results are in good agreement with them. Moreover, the existing numerical solutions for BTE are for short interval of time, while the results obtained in the paper have the potential to show the response for large intervals of time.

Furthermore, using BTE in modelling of real free vibrations of mechanical system with one-degree-of-freedom composed of a cart connected with the springs to the support and moving via linear rolling bearing block along a rail, it is observed that with certain values of the fractional order parameters, the proposed model is in good agreement with the experimental results.

In the present work, we have employed the fractional derivative definition in the sense of Caputo, but in future work we are intended to formulate BTE using the fractional ∧-derivatives to have clearer geometrical and physical basis of the model.

## Figures and Tables

**Table 1:** The identified parameters for different versions of the model.

Model	α	β	$\lambda_{0m}$	$\lambda_{1m}$	$T_{0m}$	$F_{0}\left[{\mathrm{mm}}^{2}\right]$
A	0	2	196.359	1.73345	0.436005	0.328796
B	0.9999946	1.99999	196.394	1.73192	0.436556	0.328778
C	0.5	2	224.652	092534	0.500898	0.361783
D	0.9	2	3761.75	25.1388	8.53502	0.420043
E	0	1.95	167.157	0.74288	0.370936	0.361385
F	0	1.85	120.003	0.60625	0.265951	0.51812

## Abbreviations

The following abbreviations are used in this manuscript:

FC	Fractional calculus
FDE	Fractional differential equation
BTE	Bagley–Torvik equation
